# Unveiling Silent Consequences: Impact of Pulmonary Tuberculosis on Lung Health and Functional Wellbeing after Treatment

**DOI:** 10.3390/jcm13144115

**Published:** 2024-07-14

**Authors:** Nidhi Bansal, Sumalatha Arunachala, Mohammed Kaleem Ullah, Shreedhar Kulkarni, Sukanya Ravindran, Rekha Vaddarahalli ShankaraSetty, Sowmya Malamardi, Sindaghatta Krishnarao Chaya, Komarla Sundararaja Lokesh, Ashwaghosha Parthasarathi, Bellipady Shyam Prasad Shetty, Prashanth Chikkahonnaiah, Prashant Vishwanath, Jayaraj Biligere Siddaiah, Padukudru Anand Mahesh

**Affiliations:** 1 Department of Respiratory Medicine, JSS Medical College, JSS Academy of Higher Education and Research, Mysuru 570015, India; dr.nidzz2117@gmail.com (N.B.); a.suma86@gmail.com (S.A.); shreedhar.k.kulkarni@gmail.com (S.K.); sukanya.hema29may@gmail.com (S.R.); 20457635@students.latrobe.edu.au (S.M.); chaya.sindaghatta@gmail.com (S.K.C.); lokeshpulmo@gmail.com (K.S.L.); bsjayaraj@jssuni.edu.in (J.B.S.); 2 Department of Critical Care Medicine, Adichunchanagiri Institute of Medical Sciences, Bellur 571448, India; 3 Department of Critical Care, ClearMedi Multispecialty Hospital, Mysuru 570017, India; 4 Centre for Excellence in Molecular Biology and Regenerative Medicine (A DST-FIST Supported Center), Department of Biochemistry (A DST-FIST Supported Department), JSS Medical College, JSS Academy of Higher Education and Research, Mysuru 570015, India; ka7eem@jssuni.edu.in (M.K.U.); prashantv@jssuni.edu.in (P.V.); 5 Division of Infectious Disease and Vaccinology, School of Public Health, University of California, Berkeley, CA 94720, USA; 6 School of Psychology & Public Health, College of Science Health and Engineering, La Trobe University, Melbourne 3086, Australia; 7 Rutgers University Institute for Health, Healthcare Policy, and Aging Research, The State University of New Jersey, 112 Paterson Street, New Brunswick, NJ 08901, USA; ap2320@rwjms.rutgers.edu; 8 Department of Cardiothoracic & Vascular Surgery, JSS Medical College, JSS Academy of Higher Education and Research, Mysuru 570015, India; nivishyam@gmail.com; 9 Department of Respiratory Diseases, Princess Krishnajammanni Tuberculosis and Chest Diseases Hospital, Mysuru 570002, India; prshnthcr@gmail.com

**Keywords:** pulmonary tuberculosis, post-tubercular sequelae, DLCO, chest X-ray severity, 6MWT, drop in saturation post-6MWT, spirometry

## Abstract

**Background**: Pulmonary tuberculosis (TB) remains a major public health issue in India, with high incidence and mortality. The current literature on post-TB sequelae functional defects focuses heavily on spirometry, with conflicting obstruction vs. restriction data, lacks advanced statistical analysis, and has insufficient data on diffusion limitation and functional impairment. **Objective**: This study aimed to thoroughly evaluate post-tubercular sequelae after treatment, assessing chest radiology, spirometry, diffusing capacity, and exercise capacity. **Methods**: A total of 85 patients were studied at a university teaching hospital in Mysuru. The data collected included characteristics, comorbidities, smoking history, and respiratory symptoms. The investigations included spirometry, DLCO, chest X-rays with scoring, and 6MWT. **Results**: Of the patients, 70% had abnormal X-rays post-treatment, correlating with reduced lung function. Additionally, 70% had impaired spirometry with obstructive/restrictive patterns, and 62.2% had reduced DLCO, with females at higher risk. Smoking increased the risk of sequelae. **Conclusions**: Most patients had residual radiological/lung function abnormalities post-treatment. Advanced analyses provide insights into obstructive vs. restrictive defects. Ongoing research should explore pathogenetic mechanisms and therapeutic modalities to minimize long-term post-TB disability.

## 1. Introduction

Pulmonary tuberculosis (TB) remains a global health concern, with India bearing a significant burden, accounting for 24% of global TB cases [1]. In 2020, India reported an alarming incidence rate of 188 cases per 100,000 people, causing around 480,000 deaths annually, making TB the third leading cause of years of life lost in the country [2]. Gender disparities in TB diagnosis and management are becoming more pronounced. Although both genders face significant TB morbidity and mortality risks, there are studies that have observed gender-based differences. In one study, males showed higher mortality rates [3], while other studies have shown that women experience greater disability, less improvement in health-related quality of life (HRQoL), and more years lived with disability (YLD) compared to men [4]. A study on a South Indian population 1-year post-treatment reported lower social and mental wellbeing scores among women [5].

As TB survivors increase, research on post-tuberculosis respiratory health gains importance. Despite treatment, recovered pulmonary TB individuals often face enduring issues known as post-TB sequelae [6,7]. Post-tubercular lung diseases involve diverse morphological changes: parenchymal lesions (tuberculoma, fibrosis, and cavity), airway pathology (bronchiectasis and tracheobronchial stenosis), and vascular/mediastinal lesions (arteritis, thrombosis and fibrosing mediastinitis), causing functional impairments like broncho-vascular distortions, Rasmussen’s aneurysm, and emphysema [8,9,10], reducing lung capacity and leading to respiratory deficits [6,11,12]. Bronchopleural fistula and pneumothorax can occur as complications of pulmonary TB and may require surgical intervention. Surgery may be necessary in some patients with severe complications of post-tuberculosis sequelae in the lung, such as partially or completely destroyed lung or massive hemoptysis, and these surgeries can also result in several complications, such as bronchopleural fistula, pneumothorax, respiratory failure, chest hemorrhage, heart failure, and infections [13,14,15].

Earlier research has underscored persistent lung impairment in post-TB patients post-treatment [8,9,16,17,18]. Treatment improves ventilation, but a significant deficit persists, affecting quality of life and contributing to long-term health issues and mortality [16,18,19]. Recovered TB individuals face daily activity limitations, reduced exercise tolerance, and severe impairment [18,20,21], expanding TB sequelae’s burden beyond active disease and challenging public health systems. The current literature inadequately explains functional defects in post-TB sequelae. Most of the research has extensively focused on spirometry abnormalities, and there are conflicting reports on the dominant abnormality, such as airflow obstruction versus restrictive/mixed defects [9,21,22,23]. These studies often rely on basic statistics, omitting advanced statistics to associate post-TB severity with lung function abnormalities.

Moreover, data on other sequelae, like diffusion restriction and functional limitation, are scant. Only three studies, with fewer than 300 patients combined, have globally explored these aspects [24,25,26]. However, the limitations in study design, small samples, and inadequate representation from high-TB-incidence areas compromise these studies. While functional impairments in active TB or post-therapy patients have been studied using methods like the 6 min walk test (6MWT) and diffusion restriction, overall research in this area remains inconclusive. In light of these gaps, our study seeks to comprehensively assess the prevalence and overall functional status of post-TB sequelae patients. We aimed to identify risk factors associated with abnormal chest X-rays (CXR), pulmonary physiology, and functional status and to deepen our understanding of the long-term consequences of pulmonary TB.

## 2. Materials and Methods

To unravel the intricate impact of TB on lung physiology and functional status, an observational cross-sectional study was conducted at JSS Medical College & Hospital, Mysuru, Karnataka. This study was approved by the Institutional Ethics Committee (approval number: JSS/MC/PG/5156/2020-21; date: 22 January 2021).

A total of 85 patients were enrolled in the Department of Respiratory Medicine, JSS Hospital, Mysuru, between January 2021 and June 2022, based on predefined inclusion criteria. Patients with a history of sputum-positive drug-sensitive pulmonary TB, successful completion of anti-tubercular treatment regimen HRZE, as per the National Tuberculosis Elimination program (NTEP) guidelines for 6 months [27], and the absence of contraindications for lung function tests were included. Individuals with a history of more than one episode of TB, thoracic surgery, drug-resistant pulmonary TB, and those who were HIV-positive were excluded. This study did not include any healthy controls. Comprehensive data regarding the patients’ general characteristics, comorbidities, smoking history, respiratory symptoms, lung function tests, and CXR results were collected.

To assess lung function, a series of tests was performed according to the American Thoracic Society (ATS) guidelines [28], including spirometry, pre- and post-bronchodilator spirometry, and the single-breath technique for the diffusing capacity of the lungs for carbon monoxide (DLCO). Spirometry measured the forced expiratory volume in the first second (FEV_1_), the forced vital capacity (FVC), and the FEV_1_/FVC ratio. A bronchodilator, salbutamol, was administered, and spirometry was repeated after 15 min to gauge the response. The DLCO and the total lung capacity (TLC) were recorded. Obstructive and restrictive patterns were defined based on the post-bronchodilator FEV_1_/FVC ratio and TLC values, respectively.

### 2.1. Definitions

Abnormal CXR is defined as the presence of radiographical residual changes, like fibrosis, consolidations, calcification, cavities, collapse (atelectasis), hyperinflation, pleural effusion, pleural thickening, pleural calcification, and pericardial effusion. Normal CXR is defined as no radiographical residual changes. Fibrotic scars are identified as lesions larger than 5 mm in chest X-rays, indicative of previously treated pulmonary tuberculosis in patients with a prior history of treatment for TB. These lesions are typically described as “well-defined” or “radiologically dense” and consist of nodules, linear fibrotic images with or without retraction, and bronchiectasis, usually in the upper lobes. They do not show any evidence of alveolar components or cavitations [29]. No lesion is defined as no abnormal findings on any lung fields in a chest radiograph. Cavities are defined as hollows or fluid-filled spaces in the lung tissue in a chest radiograph, indicating pathologies like abscesses or infections [30]. Consolidation is defined as solid lung areas in radiographs due to fluid or substance filling, associated with conditions such as pneumonia or tumors [30]. The smoking habit data classify patients as smokers or non-smokers. Non-smokers are defined as subjects who have never smoked. Smokers are defined as subjects who continue to smoke [31].

### 2.2. Chest X-ray Assessment

We used a scoring system developed by Báez-Saldaña et al. [22], specifically designed for the radiological severity assessment of post-TB sequelae. We did not choose the Timika score, which mainly assesses the radiological severity of active pulmonary TB [32,33]. After treatment completion, the initial assessment of the disease extent in the chest X-ray (CXR) was determined by evaluating the lung zones involved. Each lung was divided into three zones: upper zone: above the inferior wall of the aortic arch; middle zone: below the inferior wall of the aortic arch and above the inferior wall of the right inferior pulmonary vein (i.e., the hilar structures); and lower zone: below the inferior wall of the right inferior pulmonary vein (i.e., the lung bases). Disease affecting one or two zones was classified as localized, the involvement of three or four zones indicated moderate disease, and pathology present in five or all six zones was defined as extensive disease. The CXRs were categorized as normal or abnormal based on the presence of radiographic residual changes. The severity of the abnormalities was scored using a previously established system [34]. Each CXR was evaluated for the presence, distribution, and extent of lung abnormalities, including consolidation, fibrosis, lung distortion, traction bronchiectasis, pleural calcifications, and parenchymal bands. Additionally, we used another scoring system developed by Báez-Saldaña et al. [29], specifically designed to assess the radiological severity of post-TB sequelae. The lung parenchyma was divided into four quadrants, with the upper and lower lung separated at the carina. Each quadrant was scored from 0 to 5, with 0 indicating a normal radiograph and 5 indicating severe abnormalities. The score reflected the percentage of lung parenchyma involvement, with a maximum possible score of 20 for all four quadrants [22]. Severity scores < 2 in each quadrant were considered mild and scores > 2 in each quadrant indicated severe abnormalities.

### 2.3. Reader Agreement

An experienced pulmonologist and radiologist was defined as a certified specialist (readers) with more than 15 years of clinical experience. The Kappa score interpretation is as follows: <0: no agreement, 0.00–0.20: slight agreement, 0.21–0.40: fair agreement, 0.41–0.60: moderate agreement, 0.61–0.80: substantial agreement, and 0.81–1.00: almost perfect agreement [35].

### 2.4. Spirometry and DLCO

The spirometry and DLCO measurements were performed using the EasyOne Pro^®^ system from ndd Medical Technologies, Zurich, Switzerland. The forced vital capacity test was performed along with post-bronchodilator testing according to the ATS/ERS guidelines in all patients, and lung functions were considered impaired if the predicted pre-bronchodilator percentages of the FVC, FEV_1_, PEF, FEF 25-75, and FEV_1_/FVC were less than <80. DLCO measurements were also performed, and those who had a DLCO score < 80% of the predicted levels were considered to have impaired DLCO.

A post-bronchodilator FEV_1_/FVC score < 0.70 was used to categorize individuals as having obstructive airway disease, whereas if the post-bronchodilator FEV_1_/FVC score was >0.7, but the DLCO score was <80% of the predicted level, they were considered to have restrictive lung disease.

As per the numerous studies that have used interpretive algorithms to identify restrictive or obstructive patterns or to interpret spirometry findings, the following were guidelines were used:

1. Normal—an FEV_1_/FVC ratio of >70% and an FVC >80% of the predicted level;
2. Obstructive—airway obstruction was defined as an FEV_1_/FVC ratio of <70% and an FVC >80% of the predicted level;
3. Combined defects—an FVC <80% of the predicted level and an FEV_1_/FVC ratio of <0.70;
4. Restrictive—an FEV_1_/FVC ratio > 70% and an FVC < 80% of the predicted level [36].

Additionally, the functional capacity was gauged through the 6MWT as per the ATS guidelines [37], recording the distance and SpO2 changes before and after the test.

### 2.5. Statistical Analysis

The normality of the data was assessed using the Shapiro–Wilk test. If they were normally distributed, continuous variables were presented as the mean ± standard deviation. If not, they were presented as the median with their interquartile range. The categorical variables were presented as percentages. Statistical significance was assessed by the Chi-square test for categorical variables and by the Kruskal–Wallis test for continuous variables and expressed as the medians and interquartile ranges (IQRs). To extract meaningful insights from the collected data, interobserver agreements for classifying the CXR as normal or abnormal, classifying the CXR findings (cavity, fibrosis, or consolidation), and scoring the CXR severity was assessed using Cohen’s Kappa score. Additionally, for the CXR severity scoring, the mean biases and the limits of agreement (LOAs) were assessed with the Bland–Altman analysis, using the mean differences between the two scores and the 95% confident limits of the scores (LOA = mean ± 1.96 SD). The general linear model was used to analyze the association between the lung function parameters and CXR severity. Multiple linear regression and multivariate logistic regression models were utilized to ascertain the strength of association between various factors and the adjusted pulmonary function parameters. All statistical analyses were performed using jamovi-2.3.26 (v2.2.5., The jamovi Project, Sydney, Australia) statistical software. The directions of association among the lung function test variables were ascertained using Pearson’s coefficient. Two-tailed tests with *p* < 0.05 were considered statistically significant.

## 3. Results

**Figure 1:**
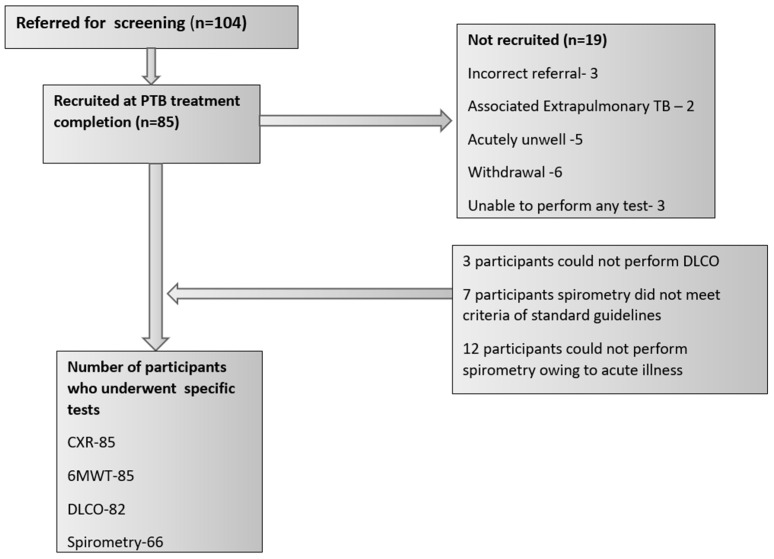
Flow diagram of participant inclusion. Note: CXR: chest X-ray; 6MWT: 6 min walk test; DLCO: diffusing capacity of the lungs for carbon monoxide.

A total of 104 individuals who had previously recovered from pulmonary tuberculosis were initially screened for eligibility. Among them, 85 individuals who fulfilled the predefined inclusion criteria were included in the study and underwent post-treatment completion CXR examinations and lung function tests to assess the presence of post-tubercular sequelae. The remaining patients who were unable to perform more than one test due to various reasons were excluded from the study (Figure 1). The median time elapsed after successful post-tubercular therapy was 12 months.

A total of 85 participants (26 females and 59 males) were included (Table 1). The mean ± SD age for the females was 37.0 ± 12.4 years, and for the males, it was 49.4 ± 16.4 years. Common co-morbidities included systemic hypertension (15.5%) and diabetes mellitus (14.3%). A significant history of smoking was reported by 42.5% of the males, and biomass fuel use was reported by 26.9% of the females. Intermittent/persistent symptoms (cough, sputum, exertional dyspnea, and dyspnea at rest) were reported by 26.9% of the females and 32.2% of the males, resulting in an overall prevalence of 30.6% (*p* = 0.626).

**Figure 2:**
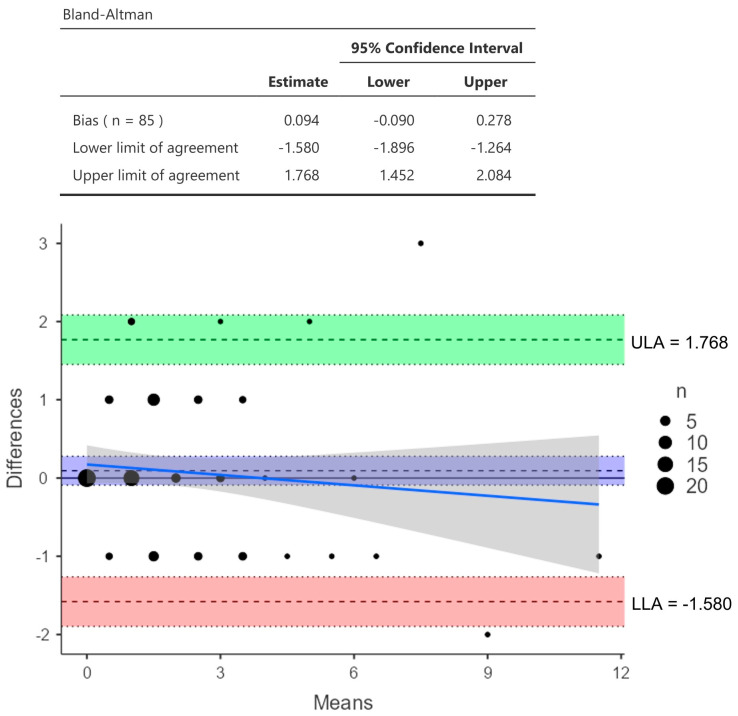
Bland−Altman plot demonstrating the level of agreement between readers 1 and 2 in chest X-ray severity scoring (X axis: the mean average numerical score between readers 1 and 2; Y axis: the difference in scores between readers 1 and 2). Horizontal lines show the mean ± 1.96 standard deviations. Note: ULA: upper limit of agreement; LLA: lower limit of agreement.

A comparison of the radiographical abnormality scores assessed by reader 1 and reader 2 showed minimal differences. The agreement between the two readers for classifying the CXR as normal and abnormal was high, as evidenced by the Cohen’s Kappa score of 0.933 (95% CI: 0.841 to 1.0), and for classifying the radiological patterns of the CXR findings (cavity, fibrosis, and consolidation), the Cohen’s Kappa score was 0.901 (95% CI: 0.792 to 1.0). For classifying the CXR severity scoring, the Cohen’s Kappa score was 0.841 (95% confidence interval (CI): 0.726 to 0.955). The Bland–Altman analysis revealed a low bias of 0.094, with 95% limits of agreement from −1.580 to 1.768 (S.D. ± 0.8539; *p* = 0.3) (Figure 2).

Among the 66 individuals who underwent spirometry assessment, 27.3% of the females and 30.2% of the males exhibited restrictive spirometry patterns (Table 2). The mean age was significantly higher in the individuals with abnormal X-ray findings (48.00 ± 16.36 years) than those with normal findings (40.27 ± 15.05 years) (*p* = 0.043). In the 6MWT, the participants with abnormal X-ray findings had a slightly lower mean percentage of predicted distance walked (85.07 ± 12.27%) compared to those with normal X-ray findings (90.96 ± 6.18%) (*p* = 0.023). The FEV_1_/FVC ratio post-bronchodilator therapy was significantly different between the normal and abnormal X-ray groups (*p* = 0.021), with a slightly lower mean value observed in the abnormal group (0.77 ± 0.14) compared to the normal group (0.82 ± 0.09). Obstructive patterns were significantly more prevalent in the abnormal X-ray group (*p* = 0.003), with no significant difference in restrictive patterns. SPO2 changes during the 6MWT significantly differed between the normal and abnormal X-ray groups (*p* = 0.004). The majority with abnormal DLCO scores had abnormal X-rays.

We observed that 26 patients had a normal chest-Xray and then further analyzed the demographic and pulmonary function test characteristics of these patients. The patients were further classified under low FVC, low FEV_1_, and low DLCO scores. In subjects with low FVC, they also had low TLC, DLCO, FEV_1_, and PEF scores. There was a female predominance in subjects with a low FEV_1_, as well as associated reductions in other spirometric parameters, and DLCO was observed. In subjects with low DLCO, in addition to reduced spirometric parameters, smoking was significantly higher (Table 3).

**Figure 3:**
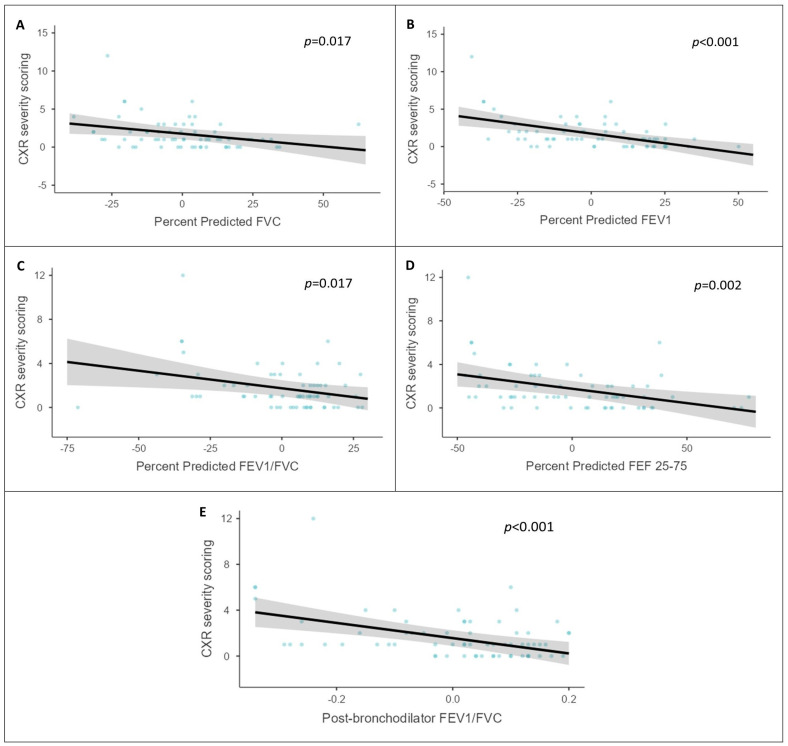
A general linear model was used to analyze the association between the lung function parameters (percentage of predicted FVC (**A**), percentage of predicted FEV_1_ (**B**), percentage of predicted FEV_1_/FVC (**C**), percentage of predicted FEF25-75 (**D**), and post-FEV_1_/FVC (**E**)) and CXR severity scoring, which showed that with increases in the CXR severity scoring, lung functions followed a decreasing trend, which is statistically significant (*p* values: percentage of predicted FEV_1_/FVC—0.017; percentage of predicted FVC—0.017; percentage of predicted FEV_1_—0.001; percentage of predicted FEF25-75%—0.002; post-bronchodilator FEV_1_/FVC—<0.001). Note: CXR: chest X-ray; FEV1: forced expiratory volume in sec 1; FVC: forced vital capacity; FEF: forced expiratory flow between 25 and 75% of vital capacity; PEF: peak expiratory flow rate.

A general linear model (Figure 3) to analyze the association between the lung function parameters (percentage of predicted FEV_1_, percentage of predicted FVC, percentage of predicted FEV_1_/FVC, percentage of predicted FEF25-75, and post-FEV_1_/FVC) and CXR severity scoring showed that with increases in CXR severity scoring, lung functions followed a decreasing trend, which is statistically significant.

Multiple linear regression analysis (Table 4) revealed the relationships between the various factors and the pulmonary function parameters (FVC, FEV_1_, FEF25-75, FEV_1_/FVC, and DLCO). After adjusting for smoking, hypertension, diabetes mellitus, drop in saturation in the 6MWT, and time elapsed after treatment (in months), the degree of radiologic abnormalities as represented by the CXR severity scoring was independently associated with percentage predicted values of FVC (a 2.5% decrease for each unit increase in CXR severity scoring; 95% CI: −4.9 to −0.16; *p* < 0.05); FEV_1_ (a 4.45% decrease for each unit increase in CXR severity scoring; 95% CI: −6.75 to −2.14; *p* < 0.001); FEF25-75%—small airway involvement (a 5.59% decrease for each unit increase in CXR severity scoring; 95% CI: −9.24 to 1.95; *p* < 0.01), and FEV_1_/FVC ratio (a 0.029% decrease for each unit increase in CXR severity scoring; 95% CI: −0.42 to −0.012; *p* < 0.001). We also observed that after adjusting for smoking, hypertension, diabetes mellitus, and time elapsed after treatment (in months), the drop in saturation in the 6MWT was independently associated with the CXR severity scoring (a 0.85 drop in saturation with each unit increase in CXR severity score; 95% CI: 0.45 to 1.25; *p* < 0.001). After adjusting for smoking, diabetes mellitus, drop in saturation in the 6MWT, time elapsed after treatment (in months), and degree of radiologic abnormalities, it was observed that patients with the presence of hypertension had a low post-bronchodilator FEV_1_/FVC ratio, suggesting potential airflow limitation.

**Figure 4:**
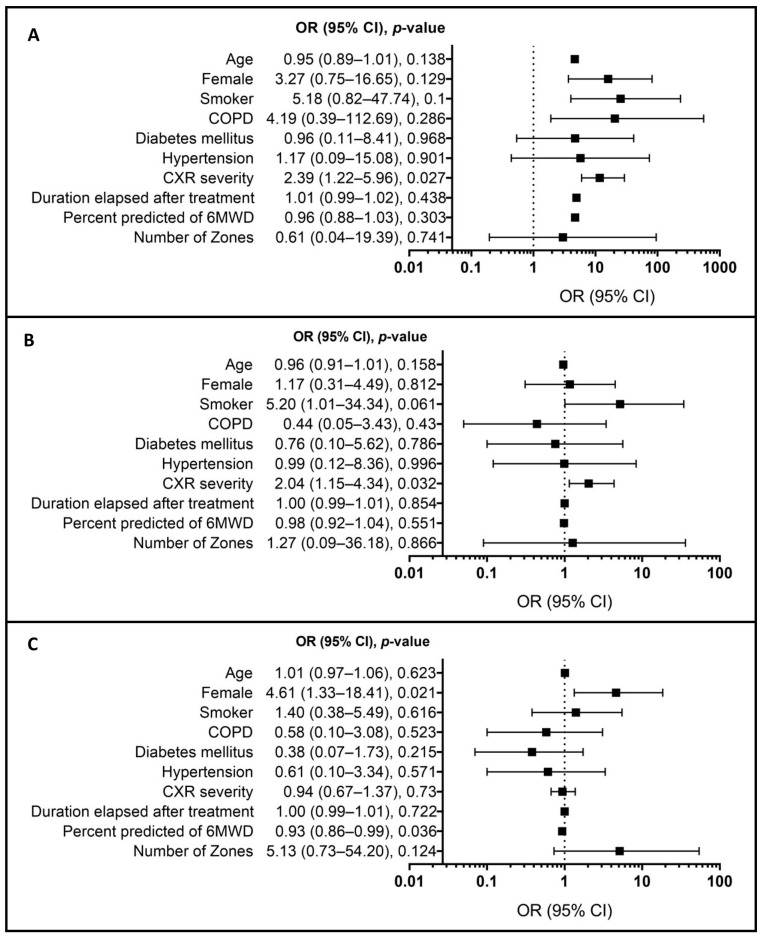
Forest plot depicting the odds ratio of different variables from a multivariate logistic regression model of low FEV_1_ (≤80) (**A**), low FVC (≤80) (**B**), and low DLCO (≤80) (**C**). Note: Male is the reference group for the female gender; CXR: chest X-ray; COPD: chronic obstructive pulmonary disease; FEV_1_: forced expiratory volume in sec 1; FVC: forced vital capacity; DLCO: diffusing capacity of the lungs for carbon monoxide 6MWD: 6 min walk test distance; OR: odds ratio; CI: confidence interval.

Figure 4 presents a forest plot depicting the odds ratio from the multivariate logistic regression model of low FEV_1_ (≤80) (Figure 4A), low FVC (≤80) (Figure 4B), and low DLCO (≤80) with other variables (Figure 4C). The findings indicate that higher CXR severity scores were significantly associated with reduced lung function, as demonstrated by the lower FEV_1_ (*p* = 0.027, OR = 2.39, 95% CI: 1.22–5.96) and FVC (*p* = 0.032, OR = 2.04, 95% CI: 1.15–4.34). The lower diffusing capacity of the lungs for carbon monoxide seen in post-tubercular patients showed preponderance to the female gender (*p* = 0.021, OR = 4.61, 95% CI: 1.33 to 18.41). No significant associations were found between the Spo2 drop in the 6MWT and any of the lung function parameters (FVC and FEV_1_).

## 4. Discussion

The results of our study shed light on the long-term effects after completion of successful medical treatment for TB and highlight the significance of continued monitoring and management of lung health in post-TB patients. The prevalence of abnormalities in lung function observed in our cohort is striking, emphasizing the need for comprehensive care beyond the completion of TB treatment. Nearly 70% of the enrolled patients displayed abnormal CXRs, with fibrosis being a prominent feature in almost half of these cases. One of the key findings of our study is the association between the CXR severity scoring and lung function abnormalities. With every unit increase in severity scoring, there was a 4.4% reduction in the percentage of predicted FEV_1_, a 2.5% reduction in the percentage of predicted FVC, a 5.5% reduction in the percentage of predicted FEF25-75 (suggesting maximal impact on small airways), and a 0.85% drop in the oxygen saturation in the 6MWT. When radiological abnormalities were present after successful completion of TB treatment, we observed greater odds of 1.79 and 2.94 for low FVC and low FEV_1_, respectively. These findings indicate the enduring impact of TB on lung tissue, leading to structural abnormalities that affect lung function. Spirometry revealed that almost 70% of the patients exhibited abnormal results. What makes these findings even more intriguing is the diverse range of patterns observed, including obstructive and restrictive patterns. In addition, 62.2% of the patients, of whom 80% were females, had abnormal DLCO, suggesting significant impairment of their diffusing capacity. Further, nearly 60% had a drop in saturation (1–4%) in the 6MWT, demonstrating diverse lung involvement in post-TB sequelae presentations, complicating the long-term consequences.

In our study, we observed that patients with abnormal CXRs experienced lower lung function as measured by spirometry, and this correlation was directly proportional to the severity of lung involvement seen in the CXRs. The link between abnormal CXRs and significant airflow limitation supports the notion that structural lesions, such as fibrosis and bronchiectasis, play a crucial role in compromising lung function. Unlike our study, Saldana et al. found that there was a 2.92% decrease in the percentage of predicted FEV_1_, with every unit increase in the CXR severity score [22]. Even though we used the same CXR severity scoring, our study found greater reduction in the FEV_1_ (a 4.45% decrease). This could be due to differences in demographics, comorbidities, proportion of smokers and pack-years, MDR-TB patients, and HIV-infected individuals, and time elapsed between the end of treatment and inclusion in the study.

Limited studies on DLCO in post-tuberculous patients have reported a decrease in DLCO levels. A study in Brazil with 36 patients found a mean DLCO ranging from 74.1% to 78.8%, while an Indian study revealed that 69% had reduced DLCO of mild to severe grade but did not show female predilection, unlike our study [23]. Interestingly, a female gender predilection for low DLCO was observed in a study of 292 patients, with 116 females having a low DLCO compared to 80 males [24]. Biomass exposure in low- and middle-income countries and late presentation may contribute to the observed gender susceptibility for greater pulmonary diffusion impairment [25]. Late presentation in post-tuberculous patients could be influenced by various factors, including social norms, anemia, malnutrition, and limited access to healthcare for women [26]. Additionally, though not evaluated in tuberculosis, sex steroids may play a role in greater interstitial involvement and delayed healing, leading to greater diffusion defects among women, as observed in previous studies that have evaluated other interstitial and chronic lung diseases [38,39,40,41,42]. Overall, more research is needed to better understand impaired DLCO in post-tuberculous patients and the influence of gender-related factors.

There was reduction in the exercise capacity of patients, especially those with CXR abnormalities, as measured by oxygen desaturation during the 6MWT and a decreased percentage of predicted distance during the 6MWT, which is similar to the findings of other studies conducted for the evaluation of exercise capacity in post-TB patients [43]. We are the first study in south-east Asian countries, to the best of our knowledge, to quantify the severity of post-tuberculosis sequelae (in terms of CXR severity scoring), and the drop in saturation in the 6MWT demonstrated a 0.85 increase in CXR severity scoring with every unit drop in SPO2 in the 6MWT.

So far, three systematic reviews and meta-analyses have been carried out for the evaluation of post-TB sequelae [44,45,46]. Most of the studies have evaluated only spirometric abnormalities, with many reporting only descriptive statistics. It is noteworthy that our study’s findings align with previous research, which has also reported a wide range of prevalence rates (41–100%) for abnormal lung function in post-TB patients [11,22,23,43,47,48,49]. Variations in demographics, comorbidities, and lung involvement severity, and differences in defining severity contribute to this variability. These factors underscore the complexity of post-TB sequelae and highlight the need for individualized patient care and tailored follow-up protocols, and the critical lack of research on identifying the risk factors for post-tubercular sequelae and additional management to prevent such life-long detrimental lung deficit. Despite confirming the profound, lifelong impact post-chemotherapy, there is a lack of guidance from the WHO and National TB programs regarding patient management. The understanding of pathogenesis and therapeutic approaches to reduce sequelae remains insufficient.

Thalidomide’s potential in mitigating lung damage by reducing TNF-alpha and suppressing lung fibrosis pathways is noteworthy [50,51]. Its role in idiopathic pulmonary fibrosis highlights its significance, suggesting clinical trials for thalidomide or other anti-fibrotic agents alongside anti-tubercular treatment. There is an urgent need for clinical trials on the co-prescription of thalidomide or other anti-fibrotic agents along with anti-tubercular treatment, as well as the identification of newer therapeutic agents to reduce post-TB sequelae.

Our study provides valuable insights into the prevalence and implications of altered lung function following successful pulmonary TB treatment. It is important to acknowledge its strengths and limitations. To the best of our knowledge, it is the first study in south-east Asia to use advanced regression techniques to quantify the association between CXR and spirometric abnormality, and a holistic assessment of lung functions was performed using spirometry, DLCO, and the 6MWT. However, the relatively small sample size, lack of CT scans, and lack of long-term follow-up remain its limitations and warrant further investigation to fully comprehend the trajectory of lung function decline in post-TB patients. Information was not available on the pre-treatment chest X-ray findings for the majority of the patients, and lung function before treatment for tuberculosis was not available for all patients.

## 5. Conclusions

Our study emphasizes that successful medical treatment for pulmonary TB does not guarantee the complete restoration of lung health. Post-TB sequelae can present as a spectrum of obstructive and restrictive lung diseases, with structural abnormalities and comorbidities playing significant roles in lung function alterations. The findings underscore the need for ongoing monitoring, individualized care, and the adoption of holistic approaches that address both the respiratory and comorbidity aspects of post-TB patients. By doing so, we can optimize the long-term outcomes and quality of life for individuals who have triumphed over TB.

## Figures and Tables

**Table 1:** Clinical characteristics of study participants stratified by gender.

		Female (*N* = 26)	Male (*N* = 59)	Total (*N* = 85)	*p* Value
Age in years	Median (IQR)	35.5 (25.9–45.1)	52.0 (36.2–61.8)	45.0 (30.0–59.0)	<0.001 *
CXR severity scoring	Median (IQR)	1.0 (0.0–2.0)	1.0 (0.0–2.8)	1.0 (0.0–2.0)	0.501 *
Duration elapsed after treatment completion (in months)	Median (IQR)	14.5 (6.0–26.0)	12.0 (5.2–46.0)	12.0 (6.0–36.0)	0.50 *
Treatment duration	Median (IQR)	6 (6.0–6.0)	6 (6.0–6.0)	6 (6.0–6.0)	0.58 *
BMI	Median (IQR)	20.0 (17.0–24.3)	21.0 (18.0–24.0)	21.0 (18.0–24.0)	0.46 *
Smoking (m)/biomass exposure (f)	n (%)	7.0 (26.9%)	25.0 (42.4%)	32.0 (37.6%)	0.18 ^†^
Hypertension	n (%)	0.0 (0.0%)	13.0 (22.0%)	13.0 (15.5%)	0.01 ^†^
Diabetes mellitus	n (%)	1.0 (3.8%)	11.0 (19.0%)	12.0 (14.3%)	0.07 ^†^
COPD	n (%)	1 (3.8)	18 (35.0)	19 (22.4)	0.007 ^†^
IHD	n (%)	0 (0)	2 (3.4)	2 (2.4)	0.342 ^†^
Persistence of symptoms	n (%)	7.0 (26.9%)	19.0 (32.2%)	26.0 (30.6%)	0.63 ^†^
Chest X-ray					0.30 ^†^
Normal	n (%)	10 (11.8%)	16 (18.8%)	26 (30.5%)	
Abnormal	n (%)	16 (18.8)	43 (50.6%)	59 (69.4%)	
No. of zones affected					0.19 ^†^
0	n (%)	10.0 (38.5%)	16.0 (27.8%)	26.0 (30.8%)	
1	n (%)	4.0 (15.4%)	20.0 (33.9%)	24.0 (28.2%)	
2	n (%)	9.0 (34.6%)	10.0 (16.9%)	19.0 (22.4%)	
3	n (%)	2.0 (7.7%)	3.0 (5.1%)	5.0 (5.9%)	
4	n (%)	1.0 (3.8%)	8.0 (13.6%)	9.0 (10.6%)	
5	n (%)	0.0 (0.0%)	1.0 (1.7%)	1.0 (1.2%)	
Spirometry (N = 66)		Female (N = 22)	Male (N = 45)	Total (N = 66)	
Restrictive	n (%)	6.0 (27.3%)	13 (30.2%)	19.0 (29.2%)	0.89 ^†^
Obstructive	n (%)	6.0 (27.3%)	17.0 (37.8%)	22 (33.8%)	0.40 ^†^
Mixed	n (%)	00 (0)	04 (9.3%)	04 (6.2%)	0.80 ^†^
Normal	n (%)	10 (45.5%)	10 (23.3%)	20 (30.8%)	0.64 ^†^
Reversibility	n (%)	3 (11%)	6 (10%)	9 (13.6%)	0.90 ^†^
DLCO (N = 82)					0.04 ^†^
>80% predicted	n (%)	05 (20.8%)	26 (44.8%)	31 (37.8%)	
<80% predicted	n (%)	19 (79.2%)	32 (55.2%)	51 (62.2%)	

* Kruskal–Wallis test, ^†^ Pearson’s Chi-Squared test. BMI: body mass index; CXR: chest X-ray; DLCO: diffusing capacity of the lungs for carbon monoxide; reversibility (FEV_1_% change post-bronchodilator).

**Table 2:** Correlations among age, CXR severity scoring, and lung function test parameters of patients stratified by normal and abnormal radiography.

Variables	X-ray Findings		Total	*p* Value
	Normal (*N* = 26)	Abnormal (*N* = 59)	Mean ± SD (*N* = 85)	
Age in years (mean ± SD)	40.27 ± 15.05	48.00 ± 16.36	45.64 ± 16.28	0.043 *
Gender				0.2962 ^†^
Female (n, %)	10 (38.5%)	16 (27.1%)	26 (30.6%)	
Male (n, %)	16 (61.5%)	43 (72.9%)	59 (69.4%)	
Treatment duration (mean ± SD)	6.1 ± 0.6	6.5 ± 1.9	6.4 ± 1.6	0.3761 *
Duration elapsed after treatment (mean ± SD)	39.4 ± 58.5	36.2 ± 57.0	37.2 ± 57.2	0.8141 *
DLCO% pred (mean ± SD)	80.69 ± 23.72	74.86 ± 23.35	76.71 ± 23.48	0.268 *
TLC % pred (mean ± SD)	88.04 ± 21.67	80.02 ± 18.91	82.56 ± 20.05	0.092 *
Distance in 6MWT % pred (mean ± SD)	90.96 ± 6.18	85.07 ± 12.27	86.87 ± 11.08	0.023 *
% Pred FVC (mean ± SD)	80.11 ± 16.11	71.21 ± 19.05	73.64 ± 18.60	0.083 *
% Pred FEV_1_ (mean ± SD)	79.3 ± 18.1	61.1 ± 19.0	66.1 ± 20.3	<0.001 *
% Pred FEV_1_/FVC Ratio (mean ± SD)	94.56 ± 21.09	87.00 ± 18.03	89.06 ± 19.05	0.153 *
% Pred FEF 25-75 (mean ± SD)	74.11 ± 27.54	48.33 ± 29.19	55.36 ± 30.80	0.002 *
% Pred PEF (mean ± SD)	92.61 ± 23.74	61.83 ± 25.23	70.23 ± 28.26	<0.001 *
FEV_1_/FVC ratio PBD (mean ± SD)	0.82 ± 0.09	0.75 ± 0.15	0.77 ± 0.14	0.021 *
FEV_1_ % change (mean ± SD)	2.84 ± 11.20	5.67 ± 10.59	4.28 ± 10.79	0.275 *
SPO2% decrease			Total	0.004 ^†^
1 (n, %)	7 (26.9%)	17 (28.8%)	24 (28.2%)	
2 (n, %)	0 (0%)	16 (27.1%)	16 (18.8%)	
3 (n, %)	0 (0%)	5 (8.5%)	5 (5.9%)	
4 (n, %)	0 (0%)	2 (3.4%)	2 (2.4%)	
5 (n, %)	0 (0%)	1 (1.7%)	1 (1.2%)	
SPIROMETRY			Total	
Obstruction (n, %)	1 (4.3%)	22 (95.7%)	23 (100%)	0.003 ^†^
Restriction (n, %)	7 (36.8%)	12 (63.2%)	19 (100%)	0.246 ^†^
CXR severity score				<0.001 ^†^
=/<2 (n, %)	0 (0)	37 (62.7%)	37 (43.5%)	
>2 (n, %)	0 (0)	14 (23.7%)	14 (16.47%)	
TLC % pred (mean ± SD)	88 ± 21.7	80 ± 18.9	82.6 ± 20.0	0.0921 *
DLCO % pred (mean ± SD)	80.7 ± 23.7	74.9 ± 23.4	76.7 ± 23.5	0.2981 *
BMI (mean ± SD)	22.7 ± 4.7	21.0 ± 5.1	21.5 ± 5.0	0.1481 *
HTN (n, %)	4 (15.4%)	9 (15.5%)	13 (15.5%)	0.9882 ^†^
COPD (n, %)	1 (3.8%)	18 (30.5%)	19 (22.4%)	0.0072 ^†^
DM (n, %)	2 (7.7%)	10 (17.2%)	12 (14.3%)	0.2482 ^†^
Smoking (n, %)	5 (19.2%)	27 (45.8%)	32 (37.6%)	0.0202 ^†^

* Kruskal–Wallis test ^†^ Pearson’s Chi-squared test. DLCO: diffusing capacity of the lungs for carbon monoxide; TLC: total lung capacity; 6MWT: 6 min walk test; PBD: post-bronchodilator; FEV_1_: forced expiratory volume in sec 1; FVC: forced vital capacity; FEF: forced expiratory flow between 25 and 75% of vital capacity; PEF: peak expiratory flow rate; % pred: percentage of predicted value; CXR: chest X-ray.

**Table 3:** Normal radiography patient characteristics and pulmonary function test results stratified by normal and abnormal FVC, FEV_1_, and DLCO values.

	FVC			FEV_1_			DLCO		
	≥80 (*N* = 16)	<80 (*N* = 10)	*p* Value	≥80 (*N* = 19)	<80 (*N* = 7)	*p* Value	≥80 (*N* = 11)	<80 (*N* = 15)	*p* Value
Age in years (mean ± SD)	40.9 ± 13.1	39.3 ± 18.5	0.8011 *	40.7 ± 14.2	39.1 ± 18.5	0.8221 *	38.4 ± 14.9	41.7 ± 15.5	0.5911 *
Gender									
Female (n, %)	5.0 (31.2%)	5.0 (50.0%)	0.3392 ^†^	5.0 (26.3%)	5.0 (71.4%)	0.0362 ^†^	2.0 (18.2%)	8.0 (53.3%)	0.0692 ^†^
Male (n, %)	11.0 (68.8%)	5.0 (50.0%)		14.0 (73.7%)	2.0 (28.6%)		9.0 (81.8%)	7.0 (46.7%)	
Treatment duration (mean ± SD)	6.0 ± 0.0	6.3 ± 0.9	0.2121 *	6.2 ± 0.7	6.0 ± 0.0	0.5551 *	6.3 ± 0.9	6.0 ± 0.0	0.2511 *
Duration elapsed after treatment (mean ± SD)	47.2 ± 61.4	26.9 ± 54.1	0.4001 *	40.8 ± 58.1	35.4 ± 64.1	0.8391 *	43.5 ± 68.0	36.4 ± 52.7	0.7681 *
Distance in 6MWT % pred (mean ± SD)	91.8 ± 5.8	89.6 ± 6.8	0.3851 *	91.7 ± 6.3	88.9 ± 5.9	0.3011 *	93.2 ± 7.5	89.3 ± 4.7	0.1191 *
SPO2% decrease									
1 (n, %)	5.0 (31.2%)	2.0 (20.0%)	0.5292 ^†^	5.0 (26.3%)	2.0 (28.6%)	0.9082 ^†^	4.0 (36.4%)	3.0 (20.0%)	0.3532 ^†^
% Pred FEV_1_ (mean ± SD)	92.1 ± 11.1	69.0 ± 16.1	0.0031 *				89.6 ± 10.8	68.9 ± 18.4	0.0101 *
% Pred FVC (mean ± SD)				88.2 ± 12.9	67.4 ± 12.3	0.0041 *	85.6 ± 12.4	74.7 ± 18.2	0.1571 *
% Pred FEV_1_/FVC (mean ± SD)							94.8 ± 30.1	94.1 ± 7.0	0.9441 *
% Pred FEF 25-75 (mean ± SD)	80.7 ± 20.8	68.9 ± 32.1	0.3841 *	85.0 ± 23.2	57.0 ± 26.4	0.0301 *	88.4 ± 24.2	59.8 ± 23.9	0.0231 *
% Pred PEF (mean ± SD)	106.9 ± 25.5	81.0 ± 15.1	0.0161 *	101.4 ± 23.6	78.7 ± 17.4	0.0441 *	103.9 ± 25.6	81.2 ± 15.9	0.0381 *
Spirometry									
Obstruction (n, %)	0.0 (0.0%)	1.0 (10.0%)	0.3572 ^†^	0.0 (0.0%)	1.0 (14.3%)	0.1972 ^†^	0.0 (0.0%)	1.0 (11.1%)	0.3032 ^†^
Restriction (n, %)	0.0 (0.0%)	7.0 (70.0%)	0.0042 ^†^	2.0 (20.0%)	5.0 (71.4%)	0.0342 ^†^	2.0 (22.2%)	5.0 (62.5%)	0.0922 ^†^
TLC % Pred (mean ± SD)	97.6 ± 22.0	72.7 ± 8.5	0.0021 *	94.1 ± 21.8	71.7 ± 10.2	0.0161 *	102.1 ± 23.1	77.7 ± 13.8	0.0031 *
DLCO % Pred (mean ± SD)	88.0 ± 25.8	69.0 ± 14.5	0.0441 *	87.6 ± 23.6	62.0 ± 10.9	0.0111 *	101.5 ± 21.4	65.4 ± 9.3	< 0.001 *
BMI (mean ± SD)	22.4 ± 3.6	23.3 ± 6.3	0.6371 *	22.6 ± 3.7	23.1 ± 7.2	0.7931 *	23.0 ± 2.8	22.5 ± 5.8	0.8091 *
HTN (n, %)	2.0 (12.5%)	2.0 (20.0%)	0.6062 ^†^	3.0 (15.8%)	1.0 (14.3%)	0.9252 ^†^	1.0 (9.1%)	3.0 (20.0%)	0.4462 ^†^
COPD (n, %)	1.0 (6.2%)	0.0 (0.0%)	0.4202 ^†^	1.0 (5.3%)	0.0 (0.0%)	0.5362 ^†^	0.0 (0.0%)	1.0 (6.7%)	0.3822 ^†^
DM (n, %)	1.0 (6.2%)	1.0 (10.0%)	0.7272 ^†^	2.0 (10.5%)	0.0 (0.0%)	0.3722 ^†^	2.0 (18.2%)	0.0 (0.0%)	0.0862 ^†^
Smoking (n, %)	2.0 (12.5%)	3.0 (30.0%)	0.2712 ^†^	2.0 (10.5%)	3.0 (42.9%)	0.0642 ^†^	0.0 (0.0%)	5.0 (33.3%)	0.0332 ^†^

* Kruskal–Wallis test ^†^ Pearson’s Chi-squared test. 6MWT: 6 min walk test; SPO2: saturation of peripheral oxygen; PBD: post-bronchodilator; FEV_1_: forced expiratory volume in 1 s; FVC: forced vital capacity; FEF: forced expiratory flow between 25 and 75% of vital capacity; PEF: peak expiratory flow rate; % pred: percentage of predicted value; TLC: total lung capacity; DLCO: diffusing capacity of the lungs for carbon monoxide; BMI: body mass index; HTN: hypertension; COPD: chronic obstructive pulmonary disease; DM: diabetes mellitus; SD: standard deviation.

**Table 4:** Multiple linear regression analysis of spirometry values and DLCO in relation to the severity scoring of radiographic abnormalities and other factors.

	FVC % Pred	FEV_1_ % Pred	FEF25-75% Pred	FEV_1_/FVC Ratio	DLCO % Pred	CXR Severity Scoring	Number of Zones
	Estimate (CI)	Estimate (CI)	Estimate (CI)	Estimate (CI)	Estimate (CI)	Estimate (CI)	Estimate (CI)
Smoker	−8.7 (−19–1.6)	−10.71 (−20.7–−0.67)	−13.97 (−29.83–1.89)	−0.053 (−0.11–0.009)	−11.45 (−23.74–0.82)	−0.08 (−1.08–0.92)	0.233 (−0.2–0.182)
Diabetes mellitus	4.96 (−7.96–17.9)	10.08 (−2.45–22.6)	17.66 (−2.17–37.5)	0.055 (−0.023–0.134)	6.12 (−9.29–21.54)	−0.32 (−1.6–0.96)	−0.024 (−0.575–0.527)
Hypertension	8.23 (−5.9–22.3)	−4.19 (−17.92–9.54)	−15.02 (−36.8–6.68)	−0.14 (−0.23–−0.062) ***	−1.75 (−17.31–13.80)	−0.93 (−2.22–0.34)	0.219 (−0.336–0.773)
CXR severity scoring	−2.5 (−4.9–−0.16) *	−4.45 (−6.75–−2.14) ***	−5.59 (−9.24–−1.95) **	−0.027 (−0.42–−0.012) ***	−2.23 (−4.94–0.48)	-	0.473 (0.376–0.570) ***
Duration elapsed after treatment (months)	−0.03 (−0.11–0.046)	−0.30 (−0.104–0.044)	−0.005 (−0.12–0.11)	−3.54 (−5.07–4.37)	0.041 (−0.05–0.134)	0.006 (−0.002–0.013)	−0 (−0004–0.003)
SPO2 drop post-6MWT	0.58 (−3.7–4.9)	−0.85 (−5.02–3.32)	−2.5 (−9.11–4.07)	−0.029 (−0.055–−0.0026) *	1.23 (−4.14–6.61)	0.85 (0.45–1.25) ***	−0.009 (−0.2–0.182)

* *p* value < 0.05; ** *p* value < 0.01; *** *p* value < 0.001; CI: confidence interval; CXR: chest X-ray; FEV_1_: forced expiratory volume in sec 1; FVC: forced vital capacity; FEF-25-75%: forced expiratory flow between 25% and 75% of vital capacity; DLCO: diffusing capacity of the lungs for carbon monoxide.

## Data Availability

All data generated or analyzed during this study are included in this published article and are available from the corresponding author upon reasonable request.
